# Integrative epigenomic analysis of differential DNA methylation in urothelial carcinoma

**DOI:** 10.1186/s13073-015-0144-4

**Published:** 2015-03-10

**Authors:** Mattias Aine, Gottfrid Sjödahl, Pontus Eriksson, Srinivas Veerla, David Lindgren, Markus Ringnér, Mattias Höglund

**Affiliations:** Division of Oncology and Pathology, Department of Clinical Sciences Lund, Lund University, Lund, Sweden; Division of Urologic Research, Department of Clinical Sciences Malmö, Lund University, Malmö, Sweden; Division of Translational Cancer Research, Department of Laboratory Medicine Lund, Lund University, Lund, Sweden

## Abstract

**Background:**

Urothelial carcinoma of the bladder (UC) is a common malignancy. Although extensive transcriptome analysis has provided insights into the gene expression patterns of this tumor type, the mechanistic underpinnings of differential methylation remain poorly understood. Multi-level genomic data may be used to profile the regulatory potential and landscape of differential methylation in cancer and gain understanding of the processes underlying epigenetic and phenotypic characteristics of tumors.

**Methods:**

We perform genome-wide DNA methylation profiling of 98 gene-expression subtyped tumors to identify between-tumor differentially methylated regions (DMRs). We integrate multi-level publically available genomic data generated by the ENCODE consortium to characterize the regulatory potential of UC DMRs.

**Results:**

We identify 5,453 between-tumor DMRs and derive four DNA methylation subgroups of UC with distinct associations to clinicopathological features and gene expression subtypes. We characterize three distinct patterns of differential methylation and use ENCODE data to show that tumor subgroup-defining DMRs display differential chromatin state, and regulatory factor binding preferences. Finally, we characterize an epigenetic switch involving the *HOXA*-genes with associations to tumor differentiation states and patient prognosis.

**Conclusions:**

Genome-wide DMR methylation patterns are reflected in the gene expression subtypes of UC. UC DMRs display three distinct methylation patterns, each associated with intrinsic features of the genome and differential regulatory factor binding preferences. Epigenetic inactivation of *HOX-*genes correlates with tumor differentiation states and may present an actionable epigenetic alteration in UC.

**Electronic supplementary material:**

The online version of this article (doi:10.1186/s13073-015-0144-4) contains supplementary material, which is available to authorized users.

## Background

Urothelial carcinoma of the bladder (UC) is one of the most common epithelial malignancies in the industrialized world and is characterized by heterogeneity in terms of the underlying molecular mechanisms. With respect to histopathology, UC can broadly be subdivided into non-muscle-invasive (NMI, stages Ta and T1) and muscle-invasive (MI, stage ≥ T2) disease. NMI disease is generally associated with a good prognosis despite frequent recurrences while MI disease has a decidedly worse prognosis [1]. Pioneering studies on phenotypic characterization of tumors using gene expression profiling have provided valuable insight into tumor biology and allowed for clinically relevant patient stratification with respect to targeted therapies [2,3]. We have previously established a molecular classification system for UC based on global gene expression patterns (Lund subtypes), and defined five major biologically distinct classes of tumors [4]. The Lund gene expression subtypes of UC include: (1) the low stage and grade Urobasal A tumors characterized by frequent *FGFR3* mutations and a good prognosis; (2) high stage and grade Urobasal B tumors that are likely progressed Urobasal A tumors; (3) Genomically Unstable tumors characterized by high tumor grade and genomic instability; (4) the poor prognosis squamous cell carcinoma-like (SCC-like) tumors characterized by expression of basal cell markers; and (5) Infiltrated tumors in which the intrinsic gene expression subtype is partially confounded by infiltrating immune and stromal cells [4,5].

Alterations in DNA methylation and chromatin modification patterns are linked features that underlie many of the phenotypic changes observed in cancer cells [6]. In recent years, the interrelations between the gene expression phenotype, the genome, as well as the DNA methylation landscape has been extensively investigated across different malignancies [7,8]. Few studies have investigated the epigenomic landscape of UC. These have highlighted aberrant expression of epigenetic writers, silencing of developmental genes, as well as topological effects on the level of histone modifications as prominent features of aggressive UC [9-11]. Importantly, a broad range of epigenetic modifiers are frequently inactivated by somatic mutations in UC, further highlighting the role of epigenetic perturbations in UC development and disease progression [12-14]. In a recent landmark publication on MI UC by The Cancer Genome Atlas project (TCGA), 34% of tumors were found to exhibit a CpG Island methylator phenotype [15], consistent with previous reports by us and others [11,16]. The TCGA study confirmed many of our findings on the gene expression subtypes of UC and validated their subtypes using our data. Although this study used mRNA expression data to stratify the tumors, it did not report on the interrelations between the gene expression phenotype and the underlying DNA methylation subtypes of UC, but instead focused on the mutation and genomic landscapes.

To address this gap and investigate the interrelations between gene expression and DNA methylation profiles, we identified differentially methylated regions (DMRs) from methylated DNA immunoprecipitation on chip (MeDIP-chip) data generated for 98 UC tumors. We show that DMR methylation patterns stratify UC tumors into clinically and biologically coherent subgroups, and provide a detailed description of associations to gene expression subtypes of UC. Our main findings were validated using TCGA data. To characterize the underlying regulatory potential of UC DMRs and show that differential methylation occurs in distinct sequence contexts, we leverage ENCODE data on chromatin states across nine cell lines [17]. Furthermore, by integrating multi-level genomic data, we are able to assign genomic context regarding chromosomal distribution, chromatin state preference, and regulatory factor (RF) binding potential to the methylation subgroup defining DMRs. These intrinsic features of the genome may have the potential to dictate the observed DNA methylation changes [18], and provide a description of the genomic processes underlying differential DNA methylation in cancer. Finally, we characterize an epigenetic switch involving the *HOXA*/*HOXB* loci, previously described in the context of stem-cell differentiation [19], and show that the state of the epigenetic switch correlates with the level of tumor differentiation and aggressiveness.

## Methods

### Tumor samples

In total 98 tumor and four macroscopically normal urothelium samples were included in the study. Detailed sample selection criteria and collected sample annotations are described in Additional file 1. Informed consent was obtained from all patients in accordance with national statutes and use of the patient material is approved by the ethical review board at Lund University. The study conformed to the Declaration of Helsinki. Gene expression data generated on Illumina HT-12 expression arrays (Illumina, San Diego, CA, USA) was available for all samples included in the study and normalized for technical biases as previously described [4]. Gene expression data processing steps as well as DMR matching procedures are described in Additional file 1.

### Methylated DNA immunoprecipitation and array hybridization

Methylated DNA immunoprecipitation [20], quality control, and purification steps, as well as PCR amplifications were performed as described in Additional file 1. Sample labeling and hybridizations to NimbleGen Human DNA Methylation 3 × 720 K CpG Island Plus RefSeq Promoter Arrays (Roche Nimblegen, Madison, WI, USA) were performed by the NimbleGen genomics facility on Iceland.

### Data filtering, normalization, and variance-based detection of DMRs

The raw probe signal intensities of the full array (Cy5 and Cy3) were extracted for each sample. Probes mapping to the 22 autosomes were kept and the remaining probes discarded from further processing. A five-step normalization scheme was applied to the data and a permutation based approach that controls for local CpG density was used to define between-tumor differentially methylated regions (Additional file 1).

### Clustering of tumor samples and DMRs

As the individual DMRs contained varying numbers of probes, we calculated the mean value of the probe scores for each DMR and tumor. We applied a bootstrap hierarchical clustering method [21] to derive stable methylation subgroups of UC tumors (Additional file 1). All calculations of sample and class enrichment and depletion with respect to clinicopathological and molecular annotations were performed using a one-versus-rest Fisher’s exact test. Survival analysis with respect to *HOX-*cluster subtypes was performed using the logrank test on the entire cohort with disease specific survival as endpoint and was not corrected for clinicopathological variables or treatment.

### Annotation of genomic features to DMRs

RefSeq tracks for genes, CpG-islands, chromatin tracks for nine ENCODE cell lines [17], as well as the RepeatMasker track for hg18 were downloaded from the UCSC genome browser. Evolutionarily constrained elements throughout the genome, defined using the GERP algorithm [22], were obtained from the Sidow-lab web page. The MSigDB v3.1 database was downloaded from the GSEA web page. All processing steps related to genomic feature annotation of UC DMRs are described in Additional file 1.

### TCGA data validation

For all samples included in the study (N = 234), data on DNA methylation (Illumina Infinium HM450 arrays), somatic variants (exome sequencing), and gene-level RNA sequencing were downloaded from the TCGA ftp server. We also obtained methylation data for 21 adjacent normal samples from TCGA. All raw file names are listed in Additional file 2: Table S1. The normalized gene-level expression estimates were processed by adding the constant 1 to all expression estimates followed by log2 transformation and median centering. The methylation data matrix was filtered for all probes with a SNP-annotation or a missing value in any tumor sample (final N = 322,425 probes). For the somatic mutation data, silent variants were filtered out and the mutation status of each gene was dichotomized on the sample level. For details on data processing steps, see Additional file 1.

### Processing and analysis of ENCODE data

For compatibility reasons the UC DMRs were mapped to the hg19 build of the human genome using the UCSC liftover tool and resulted in a successful conversion for all but three DMRs (N = 5,450). Data on regulatory factor ChIP-seq peak calls as well as DNaseI-sites generated by the ENCODE consortium were obtained through the UCSC genome browser and processed as described in Additional file 1.

### Statistical analyses and data visualization

All data manipulation, normalization, and calculation steps were carried out in the R statistical programing environment. All data visualization was produced using base graphics in R, and the UCSC genome browser.

### Data access

The data generated through this study have been deposited into the Gene Expression Omnibus (GEO) under the accession number GSE58256. The previously published gene expression data are available under the accession number GSE32894. The data generated by TCGA are available through the project ftp site and ENCODE data through the UCSC genome browser.

## Results

### Tumor-intrinsic DMR methylation patterns define four UC subgroups

We profiled 98 UC samples representative of the full clinicopathological spectrum using MeDIP followed by hybridization to Nimblegen 3 × 720 K RefSeq promoter and CpG Island (CGI) arrays to identify between-tumor differentially methylated regions (DMRs). We identified 5,453 high-confidence regions throughout all autosomes (Median size 780 bp, range 500 to 4,610 bp; Methods, Additional file 3: Table S2).

To define subgroups of UC based on their DMR methylation profiles, we used the 25% most varying DMRs (N = 1,363) and a bootstrap hierarchical clustering approach for tumor sample clustering [21]. The approach yielded four robust methylation subgroups (subgroups 1 to 4, Methods) which differed with respect to both clinical (pathological stage and grade) and molecular characteristics, including the Lund gene expression and the Lauss *et al.* [11] DNA methylation epitypes (epitype A-C) of UC as well as *FGFR3* and *TP53* mutation frequencies (Table 1) [4,11].

**Table 1:** **Patient characteristics for all 98 UC samples included in the study**

**Patient characteristics**			**Subgroup 1**	**Subgroup 2**	**Subgroup 3**	**Subgroup 4**
		**Total**	**N**	**N**	**N**	**N**
Samples	N	98	18	21	24	35
Gender	Male	69	12	16	19	22
	Female	29	6	5	5	13
WHO 1999 Stage	Ta	45	16	14	5	10
	T1	25	2	5	9	9
	MI	27	0	1	10	16
	Tx	1	0	1	0	0
WHO 1999 Grade	Grade 1	19	11	5	1	2
	Grade 2	32	7	11	4	10
	Grade 3	47	0	5	19	23
TP53 mutation	Mutation	29	0	4	11	14
	Wild type	69	18	17	13	21
FGFR3 mutation	Mutation	34	12	11	1	10
	Wild type	64	6	10	23	25
Lund subtype	Urobasal A	44	18	15	2	9
	Urobasal B	12	0	5	2	5
	Genom Unst	29	0	0	17	12
	SCC-like	13	0	1	3	9
Lauss epitype	A	17	9	6	0	2
	B	9	1	1	0	7
	C	17	0	1	11	5
	D	7	0	1	0	6
	NA^a^	48	8	12	13	15
Age (years)	Median	71.7	68.5	70.3	70.6	75.4
	Range	(43.5-94.8)	(43.9-84.3)	(49.7-93.0)	(59.0-94.8)	(43.5-93.5)

^a^Samples not included in the Lauss *et al.* [11] study.

Subgroups 1 and 2 were enriched for stage Ta tumors (*P* <5 × 10^-5^ and *P* = 0.047, respectively, Fisher’s exact test) and tumors of lower pathological grade (subgroup 1 for grade 1, *P* <2 × 10^-5^ and subgroup 2 for grade 2, *P* = 0.038). Both groups exhibited enrichment for the Urobasal A gene expression subtype (*P* <6 × 10^-8^ and *P* = 0.0071, respectively) and the previously defined epitype A (*P* <8 × 10^-5^ and *P* = 0.047). Moreover, subgroup 1 was enriched for activating *FGFR3* mutations (*P* = 0.0026). Subgroup 3 was enriched for pathological grade 3 tumors (*P* = 0.0008) and exhibited a strong association with the Genomically Unstable gene expression subtype (*P* <2 × 10^-6^) and epitype C tumors (*P* <4 × 10^-7^). This subgroup did not differ significantly with respect to *TP53* mutation frequency, but was depleted of *FGFR3* mutations (1 of 24 tumors, *P* = 0.0002). Subgroup 4 was characterized by an association to MI (*P* = 0.0043) and pathological grade 3 (*P* = 0.012) disease, enriched for the poor prognosis SCC-like gene expression subtype (*P* = 0.011) and also included the majority (78%) of epitype B samples (*P* = 0.021). Subgroup 4 did not differ significantly from the other subgroups with respect to *FGFR3* or *TP53* mutation status. The identified methylation subgroups therefore correspond well to previously established molecular and epigenetic subtypes, as well as to pathologically, clinically, and genetically distinct classes of tumors.

### The genomic characteristics of subgroup defining DMRs

**Figure 1:**
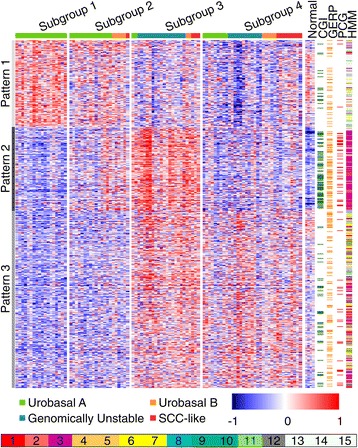
**Patterns of DNA methylation across UC methylation subgroups.** The methylation levels for subgroup-specific DMRs are shown for 98 UC samples (columns) divided into the four methylation subgroups. The subgroup-specific DMRs (rows) are grouped into patterns 1 to 3. Relative DNA methylation levels are shown as a heat map pseudo-colored blue (unmethylated) to red (methylated). The Lund gene expression subtypes of the UC samples are shown at the top of the heat map. Characteristics of DMRs are shown in five panels to the right of the heatmap: DMR methylation profiles in four normal urothelium samples (Normal); CpG Island (CGI; green); NCEC overlap (GERP; yellow); Lee *et al.* 2006 Polycomb targets (PCG; red); chromatin states according to Ernst *et al.* [17] (HMM; 1, ‘Active promoter’; 2, ‘Weak promoter’; 3, ‘Inactive/poised promoter’; 4 to 5, ‘Strong enhancer’; 6 to 7, ‘Weak enhancer’; 8, ‘Insulator’; 9, ‘Transcriptional transition’; 10, ‘Trancriptional elongation’; 11, ‘Weak transcribed’; 12, ‘Polycomb repressed’; 13, ‘Heterochromatin/low signal’; 14 to 15, ‘Repetitive/CNV’).

We then applied ANOVA on the full set of 5,453 UC DMRs to identify regions with subgroup-specific methylation patterns. ANOVA significant DMRs (N = 2,697, *P* <0.05, FDR corrected) exhibited a higher median CpG density compared to DMRs without subgroup-specific methylation patterns (median = 0.022 and 0.011 CpG/bp, respectively, *P* <7 × 10^-82^, Mann-Whitney U test, Additional file 4: Figure S1A). We observed a significant difference in CpG density between UC DMRs that are hyper- (high CpG density) and hypomethylated (low CpG density) in tumor samples compared to normal urothelium (Additional file 4: Figure S1B). Hierarchical clustering of the subgroup specific DMRs revealed three main methylation patterns across the data (Figure 1 and Additional file 5: Figure S2A to C), hereafter referred to as methylation pattern 1 (672 DMRs), 2 (650 DMRs), and 3 (1,375 DMRs).

Pattern 1 DMRs showed a gradual drop in methylation levels with increasing tumor grade (r = -0.46, *P* = 2 × 10^-6^) and across the four methylation subgroups. These DMRs were predominantly located in CpG-poor regions of the genome as measured by CpG/bp (median = 0.014 CpG/bp) as well as by depletion of overlaps with CGIs (*P* <2 × 10^-10^, Fisher’s exact test, Figure 1). As evolutionary conservation predicts functionality, we also calculated the basewise overlap of each DMR with non-coding evolutionarily conserved (NCEC) elements defined by the GERP algorithm [22] (Methods). Pattern 1 DMRs exhibited a significant depletion of NCEC element overlaps (*P* <4 × 10^-5^, Figure 1). We also quantified the basewise overlap of DMRs with H1ESC chromatin states and assigned each DMR a consensus chromatin state by majority vote (Figure 1). The 15 chromatin states were derived by Ernst *et al.* [17] using data on genome-wide histone modification patterns as well as CCCTC-binding factor (CTCF) occupancy. The chromatin states accurately captured the regulatory potential associated with genomic segments and were named according to associated sequence functions, for example, ‘Active promoter’ for transcription start site (TSS) related sequences or ‘Poised promoter’ for regions displaying bivalent [23] marks. Pattern 1 DMRs showed significant depletion of marks associated with functional sequences such as ‘Active promoter’, ‘Poised promoter’, and ‘Weak/poised enhancer’, but were instead strongly enriched for the ‘Heterochromatin/low signal’ state (*P* <1 × 10^-80^).

Pattern 2 DMR methylation stood out as the defining characteristic of subgroup 3 tumors (Figure 1). These DMRs were enriched for high CpG content (median = 0.048 vs. 0.014 CpG/bp, *P* <4 × 10^-143^, Mann-Whitney U test), CGI (*P* <4 × 10^-52^, Fisher’s exact test) and CGI-shore overlaps (*P* <2 × 10^-14^). We observed a high degree of overlap between pattern 2 DMRs and NCEC elements (*P* <5 × 10^-21^). With respect to H1ESC chromatin states, pattern 2 DMRs exhibited a robust enrichment for overlaps with the ‘Inactive/poised promoter’ state (*P* <1 × 10^-205^). In order to further substantiate the link between pattern 2 methylation and the ‘Inactive/poised promoter’ chromatin state in embryonic stem cells, we also mapped DMR overlaps with the H9ESC-derived polycomb repressive complex 2 (PRC2) target signature characterized by Enhancer of Zeste Homolog 2 (EZH2), SUZ12 Polycomb Repressive Complex 2 Subunit (SUZ12), and Embryonic Ectoderm Development (EED) binding [24], and again found high levels of enrichment among pattern 2 DMRs (*P* <8 × 10^-61^, Figure 1).

Pattern 3 DMRs displayed an inverse correlation with pattern 1 DMRs, that is, an increasing level of methylation with higher tumor grade. DMRs exhibiting pattern 3 methylation were depleted of CGI- and enriched for CGI-shore overlaps (*P* <2 × 10^-14^ and *P* <4 × 10^-60^, respectively). Pattern 3 DMRs showed a weak enrichment for NCEC sequence overlaps (*P* = 0.0074). Moreover, pattern 3 DMRs showed an association with functional regions such as ‘Active promoter’ (*P* = 0.033), ‘Weak promoter’ (*P* <7 × 10^-16^) and ‘Inactive/poised promoter’ (*P* = 0.0002), while being depleted of overlaps with the ‘Heterochromatin/low signal’ state (*P* <6 × 10^-50^). Pattern 3 DMRs were enriched for both ‘Strong enhancer’ as well as ‘Weak/poised enhancer’ states (*P* <3 × 10^-5^ and *P* <3 × 10^-9^, respectively).

Hypomethylation of DNA has been shown to occur at LINE1 and LTR elements in immortalized fibroblasts and at chromosome ends in a subset of glioblastoma tumors with potential implications for genome function [25,26]. To explore this further, we quantified the local (2 kb window) LINE1 and LTR element content and mapped the distance to the nearest chromosome end for each DMR. In total 1,523 (28%) of all DMR regions contained LINE1 or LTR repetitive elements and the median overlap, when present, was 326 bp. When considering only the subgroup-specific DMRs; pattern 1 DMRs showed significant enrichment of repetitive sequence overlaps (*P* <3 × 10^-11^), while pattern 2 DMRs were strongly depleted of local repetitive element overlaps (*P* <4 × 10^-18^, Additional file 6: Figure S3A). Pattern 1 DMRs exhibited enrichment in subtelomeric regions of the genome measured as distance to nearest chromosome end (*P* <3 × 10^-34^, Kruskal-Wallis test, Additional file 5: Figure S3B), or relative enrichment of elements within the first or last 5 Mb of chromosomes (*P* <2 × 10^-32^, Fisher’s exact test, Additional file 5: Figure S3C). Taken together, by integrating data from multiple levels, we show that subgroup-specific differential DNA methylation occurs in distinct genomic contexts.

### The biology of DMR-associated genes and correlations to gene expression

Overall we were able to match 3,685 (68%) of all DMRs to Illumina microarray gene expression data [4] (Methods). We used a resampling-based method to derive empirical significance thresholds for correlations between DMR methylation and gene expression (Methods). In total, 708 DMR-gene correlation coefficients, mapping to 668 unique DMRs, were found to be significant (18% of all gene expression matched DMRs). Among the 708 significant DMR-gene correlations, 477 (67%) were negative. When only considering gene expression matched DMRs, 16.2% of the pattern 1 DMRs and 14.2% of pattern 2 DMRs exhibited significant correlations to gene expression. By contrast, 31.4% of pattern 3 DMRs exhibited significant correlations to gene expression. We observed a substantial difference between pattern 2 and pattern 3 DMRs with respect to methylation-gene expression correlations in loci marked by the ‘Inactive/Poised Promoter’ state in H1ESC. Pattern 3 ‘Inactive/Poised Promoter’ state DMRs were four times more likely to be significantly associated with gene expression than were pattern 2 ‘Inactive/Poised Promoter’ state DMRs (37.9% vs. 9.4%, *P* <5 × 10^-13^) compared to approximately two-fold when considering pattern 2 and 3 DMRs irrespective of chromatin state in H1ESC. An analysis of biological themes related to the methylation patterns observed across UC DMRs was conducted using the signatures contained in the MSigDB v3.1 database (Methods) and highlighted differential enrichment of stem cell and developmental gene related signatures [27,28] (Additional file 7: Table S3). Taken together, pattern 3 DMR methylation highlights regions of active transcriptional regulation in UC, whereas pattern 2 DMRs accrue methylation in a subset of tumors with an absence of corresponding effects on gene expression patterns. Pattern 1 DMR methylation did not affect coordinated and biologically relevant gene expression programs.

### Validation of DMR methylation patterns using TCGA data

**Figure 2:**
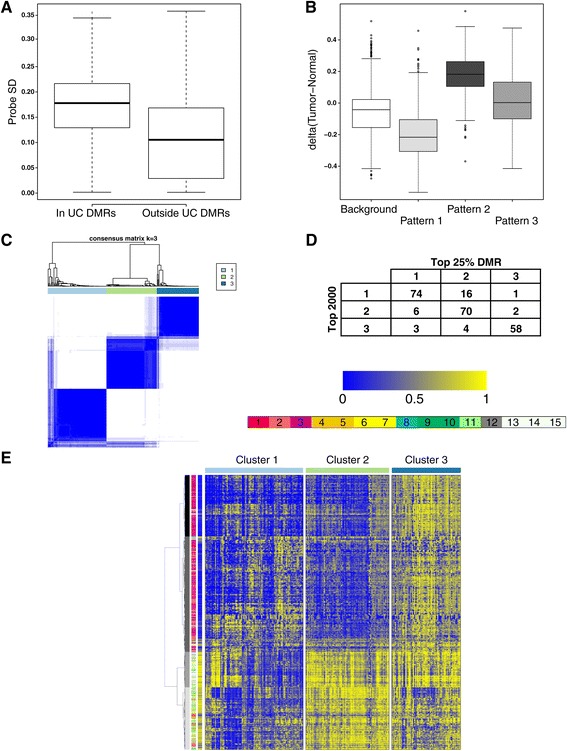
**External validation in TCGA data. (A)** Standard deviation of probes overlapping UC DMRs and those that are outside of UC DMRs. **(B)** Differences in mean methylation levels between tumors and adjacent histologically normal tissue stratified by methylation patterns. **(C)** Heatmap of co-clustering frequencies of tumors derived by K-means consensus clustering of the 2,000 most variable probes in the TCGA data. **(D)** Tumor subgroupings derived using K-means clustering on the 25% most varying DMR-overlapping probes or the 2,000 most variable probes. **(E)** Hierarchical clustering of probes and heat map visualization of the three methylation clusters. The plot shows four main clusters of differentially methylated probes (leftmost annotation bar) and the associated chromatin states in H1ESC (middle bar, states as in Figure 1). The average methylation level in adjacent normal bladder tissue is indicated in the rightmost bar.

We sought to validate the main observed methylation patterns in independent data generated by TCGA. We obtained methylation data for 234 MI tumors as well as 21 adjacent normal samples. We confirmed the high variance nature of UC DMR methylation by comparing the standard deviation of DMR overlapping (N = 9,969) and non-overlapping probes (N = 308,871). Probes within DMRs exhibited substantially higher variability in the external dataset (Figure 2A, *P* <2.2 × 10^-16^, Mann-Whitney U test).

We extracted the most variable probe for all covered UC DMRs (3,361 probes) and investigated the average methylation state in tumors versus adjacent normal tissue (21 samples). These results confirm the platform (Illumina vs. Nimblegen) and cohort (NMI and MI vs. MI only) independent nature of our findings with respect to the three DMR patterns (Figure 2B), that is, demethylation of pattern 1 DMR overlapping probes, hypermethylation of pattern 2 DMR overlapping probes, as well as elevated methylation levels of pattern 3 DMR overlapping probes.

To evaluate whether UC DMR methylation is representative of the overall variation structure of the full dataset, we clustered the top 25% most varying DMR-overlapping probes (N = 841 of 3,361) as well as the 2,000 most varying probes from the full platform using K-means consensus clustering [29]. We were able to derive stable subgroups using both datasets and found that a three-group split captured well the main structure of the data (Figure 2C). Despite a limited overlap between the two probe sets (N = 279), the respective tumor co-clustering results were highly concordant (*P* <3 × 10^-65^, Chi-square test, Figure 2D). These results confirm that the UC DMRs defined in our MeDIP-experiment capture the main variation structure in an exclusively MI cohort generated on a different platform.

Clustered heatmap visualization of the 2,000 most varying probes in relation to consensus cluster subgroups derived using the same data revealed two opposing patterns driving sample stratification: *de novo* methylation of high CpG density regions and demethylation of CpGs in lower density regions. The major methylation patterns were robustly associated with H1ESC chromatin states and the UC DMR methylation patterns defined in the discovery MeDIP-set. The row (probe) dendrogram indicated four major branches with 448, 840, 258, and 454 probes, respectively (Figure 2E). We tested each of the four branches for skewness with regard to UC DMR overlaps. We found that two branches (N = 840 and 454 probes) marked by H1ESC active/weak/poised promoter (states 1 to 3) or transcription and enhancer (states 9 to 11 and 6 to 7) associated states respectively, were enriched for pattern 3 DMRs (*P* <0.0003 and *P* <0.0007, respectively (Fisher’s exact test). A branch (N = 448) that was dominated by probes in regions with bivalent marks (state 3) in H1ESC displayed enrichment of pattern 2 DMRs (*P* <5.4 × 10^-21^). Finally a H1ESC ‘heterochromatin/low signal’-dominated branch (N = 258 probes) showed a significant enrichment of pattern 1 DMR overlaps (*P* <9.6 × 10^-13^), confirming the existence of at least three distinct and pervasive sequence contexts that are differentially methylated in UC.

Thus we were able to validate our main findings on differential methylation in an independent data-set of 234 MI UC tumors run on Illumina Methylation 450 K arrays by TCGA. We show that probes overlapping UC DMRs display higher variance than non-overlapping probes, that probes overlapping pattern 1 to 3 DMRs exhibit the same directionality of differential methylation with respect to adjacent normal tissue, that UC DMR overlapping probes capture the variation structure of the data as a whole, and that unsupervised hierarchical clustering of probes identifies pattern 1 to 3 methylation in the validation data.

### Chromatin state characterization of UC DMRs across ENCODE cell lines

**Figure 3:**
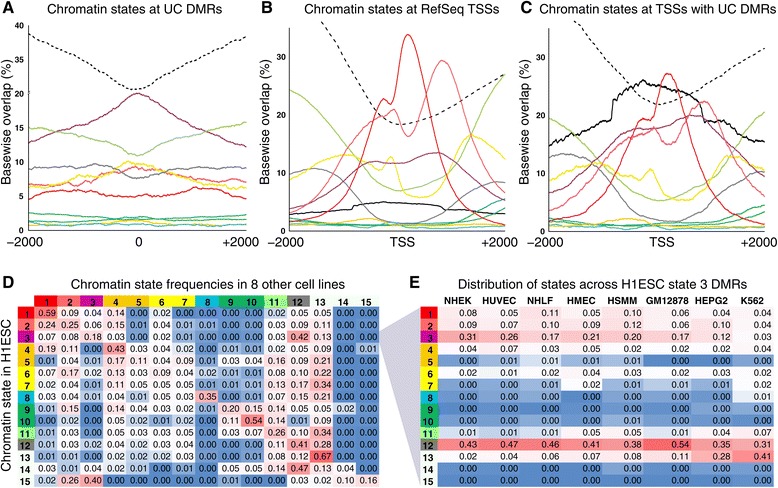
**Chromatin state characterization of UC DMRs. (A)** Frequency of H1ESC chromatin states for all UC DMRs (N = 5,453) calculated per base across 4 kb windows centered on each DMR midpoint. **(B)** Frequency of H1ESC chromatin states for all human RefSeq promoters calculated per base across 4 kb windows centered on each RefSeq TSS (N = 27,397). The corresponding frequency of UC DMR overlaps is also shown (solid black line). **(C)** Frequency of H1ESC chromatin states from RefSeqs having a UC DMR in the 4 kb window centered on its TSS (N = 5,290). **(D)** Chromatin states for H1ESC and eight additional cell lines (listed in panel E; Ernst *et al.* [17]). For UC DMRs in a H1ESC chromatin state, the frequencies of the typical chromatin state in the eight other cell types are shown. **(E)** For UC DMRs in the ‘Inactive/poised promoter’ state in H1ESC, the frequencies of other chromatin states are shown for the eight additional cell types. The 15 Chromatin states are color coded as in Figure 1 (chromatin states 13 to 15 are represented by a dashed line in panels A to C).

To further characterize the regulatory potential associated with UC DMRs, we mapped the overlaps of H1ESC chromatin states UC DMRs. The distribution of chromatin states at UC DMRs exhibited a clear pattern of decreasing ‘Heterochromatin/low signal’ marks and a corresponding increase in states associated with promoter and gene regulatory regions towards DMR midpoints (Figure 3A). The ‘Inactive/poised promoter’ as well as ‘Weak promoter’ and ‘Weak enhancer’ states showed increased frequencies towards the midpoint of DMRs while the ‘Weak transcription’, ‘Polycomb repressed’, and ‘Active promoter’ state overlaps exhibited decreases.

We also mapped the chromatin context at autosomal RefSeq transcription start sites in aggregate (TSSs, Figure 3B) and in regions with UC DMRs (Figure 3C). Average chromatin state frequencies differed between promoters with DMRs and all autosomal RefSeq promoters (Figure 3B and C), in that an increase of ‘Inactive/poised promoter’ marks, mainly at the expense of the ‘Active promoter’ mark, was observed in areas having DMR overlaps. The average pattern of DMR overlaps around TSSs revealed a steady increase towards the TSS and a clear plateau covering approximately -700 to +700 relative to the TSS (Figure 3C).

Ernst *et al.* [17] also derived chromatin state maps for eight additional cell lines representative of different embryonal lineages and differentiation states. We mapped alternate chromatin states in these cell lines onto UC DMRs, yielding eight transition states for each UC DMR (Methods). The co-occurrence frequencies of alternate states in relation to the H1ESC states are shown in Figure 3D and highlight the relative stability of a subset of chromatin states, for example, ‘Active promoter’ and ‘Heterochromatin/low signal’, as well as the dynamic nature of others such as the ‘Weak enhancer’ and ‘Inactive/poised promoter’. As UC DMRs were frequently marked by the ‘Inactive/poised promoter’ state in embryonic stem cells (Figure 3A), we investigated the range of states of H1ESC ‘Inactive/poised promoter’-marked loci in the additional eight cell lines. The analysis revealed that most UC DMRs marked by ‘Inactive/poised promoter’ marks resolve to a monovalent, predominantly H3K27Me3 marked (‘Polycomb repressed’) state across all eight cell lines (Figure 3E), consistent with observations in stem-cell differentiation [23,27]. In contrast to the other cell lines, the two cancer-derived cell lines (HepG2 and K562) frequently exhibit heterochromatin marks at H1ESC bivalent loci (Figure 3E). Our results highlight the dynamic nature of chromatin modifications across cell types and differentiation states while providing independent evidence of regulatory function for UC DMRs.

### Regulatory factor occupancy of UC DMRs

**Figure 4:**
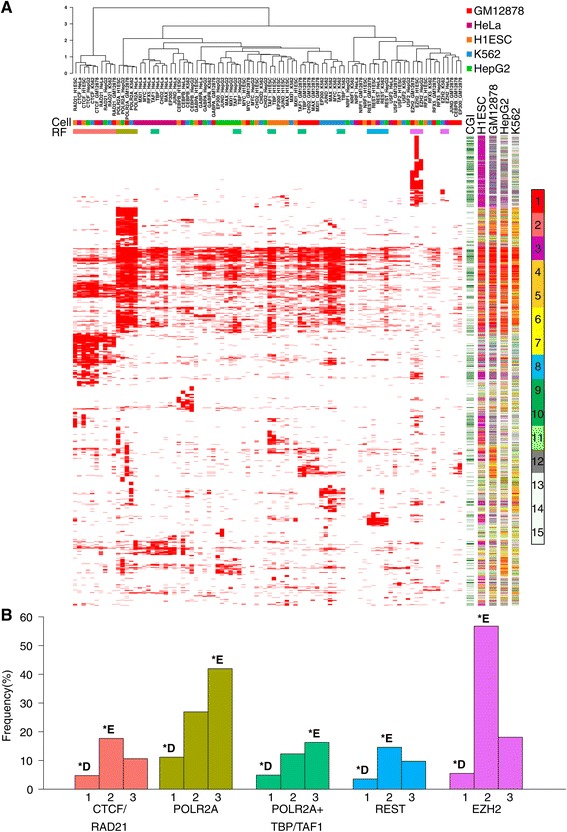
**Regulatory factor occupancy at UC DMRs. (A)** Ninety RF cell type combinations are clustered based on the occupancy of the RF in the respective cell type at UC DMRs. Binary overlaps between UC DMRs and 18 different RFs across the five cell types are shown in red. The top color bars indicate different cell types, and highlight CTCF/RAD21 binding (pink), POLR2A (olive), TAF1/TBP (turquoise), REST (light blue), and EZH2 (purple) binding, respectively. Characteristics of DMRs are shown in five panels to the right of the heatmap: CpG Island (CGI; green); H1ESC, GM12878, HEPG2, K562, chromatin states for four cell types (see color bar). The clustering highlights the cell type independent binding patterns at UC DMRs of RFs such as CTCF, RAD21 and POLR2A, as well as the similar but cell-type specific binding patterns of TBP and TAF1 across all cell lines. **(B)** The frequency of UC DMRs with RF binding is shown for selected RFs and combinations of RFs for each methylation pattern (1 to 3). Strong depletion (*D) or enrichment (*E) of RF binding is indicated (all *P* <4 × 10^-6^, Fisher’s exact test), the regulatory factor frequency bars are color coded as the factors in (A).

We then utilized ChIP-seq data generated by the ENCODE consortium [30], to gain further insights into the basic gene regulatory and genomic context of regions associated with methylation changes in UC and to analyze our data from a regulatory factor (RF)-based perspective. We focused on the five cell lines for which the largest amount of data on RFs was available and restricted our analysis to UC DMRs (Methods). We extracted a core set of 18 RFs for which data were available in all five cell lines (90 RF-cell tracks in total) and constructed a binary matrix of DMR-ChIP-seq peak overlaps, which we subsequently clustered (Figure 4A, Methods). Fifty-five percent of all UC DMRs had at least one overlapping ChIP-seq peak. While 88% of pattern 2 and 71% of pattern 3 DMRs had at least one overlapping ChIP-seq peak, this was only the case for 27% of pattern 1 DMRs.

A subset of UC DMRs clustered together mainly due to the influence of CTCF and RAD21 homolog (RAD21) binding (Figure 4A). CTCF/RAD21 co-binding may implicate these regions as functional elements in a process such as cohesin recruitment [31]. Specific co-occurrence of CTCF and RAD21 peaks at UC DMRs was assessed by identifying all instances in which a DMR overlapped both CTCF and RAD21 peaks in at least one cell line (N = 516). Pattern 2 DMRs exhibited significant enrichment for CTCF-RAD21 co-binding (*P* <3 × 10^-12^, Fisher’s exact test, Figure 4B), and the enrichment was mainly attributable to the H1ESC cell-line.

In total, 1,548 DMRs (28.4%) were bound by RNA polymerase 2 (POLR2A) in at least one cell line. POLR2A binding exhibited three main patterns across the UC DMRs: (1) ubiquitous and exclusive POLR2A binding characterized by enhancer states in all cell lines; (2) ubiquitous POLR2A binding with active promoter states and frequent RF binding across multiple cell lines; and (3) patterns of POLR2A co-binding with RFs and enhancer/promoter states in a cell type specific context (Figure 4A). Nearly 40% (37.3%) of the DMRs overlapping POLR2A sites exhibited pattern 3 methylation (*P* <3 × 10^-36^, Figure 4B), while at the other extreme, pattern 1 DMRs were strongly depleted of POLR2A binding (*P* <6 × 10^-30^, Figure 4B). The transcription-associated RFs TATA-binding protein (TBP) and Transcription initiation factor TFIID subunit 1 (TAF1) always clustered together in a cell-type specific manner (Figure 4A). For each of the five cell-lines, co-binding of all three transcription-associated RFs in any of the cell lines was recorded and enrichment statistics were calculated for the three DMR categories. Only pattern 3 DMRs exhibited significant enrichment for regions bound by all three factors (*P* <3 × 10^-8^, Figure 4B).

For sites bound by the RE1-Silencing Transcription Factor (REST) in any of the cell lines (N = 511), pattern 2 DMRs exhibited significant enrichment for overlaps (*P* <4 × 10^-6^, Figure 4C). The enrichment was further accentuated in the subset of DMRs that were bound by REST in all five cell lines (N = 60, *P* <3 × 10^-8^). A large number of UC DMRs exhibited EZH2 binding, consistent with chromatin state annotations showing frequent overlaps with bivalent state marked regions in H1ESC (Figure 4A). As expected pattern 1 DMRs were strongly depleted of EZH2 binding (*P* <2 × 10^-17^, Figure 4B) while pattern 2 DMRs exhibited robust enrichment (*P* <7 × 10^-155^, Figure 4B). In addition to establishing the differential binding landscape of RFs with respect to UC DMRs, we highlight the connection between RF binding and chromatin states in both a cell-type specific and independent context. Our results provide evidence in favor of multiple genomic processes underlying the DMR methylation patterns 1 to 3 observed across UC tumors, and implicate pattern 1 DMRs as infrequent sites of RF binding, pattern 2 DMRs as frequent sites of CTCF/RAD21 as well as EZH2 binding, and pattern 3 DMRs as sites of frequent POLR2A occupancy.

### Spatial patterns of regulatory factor binding at UC DMRs

**Figure 5:**
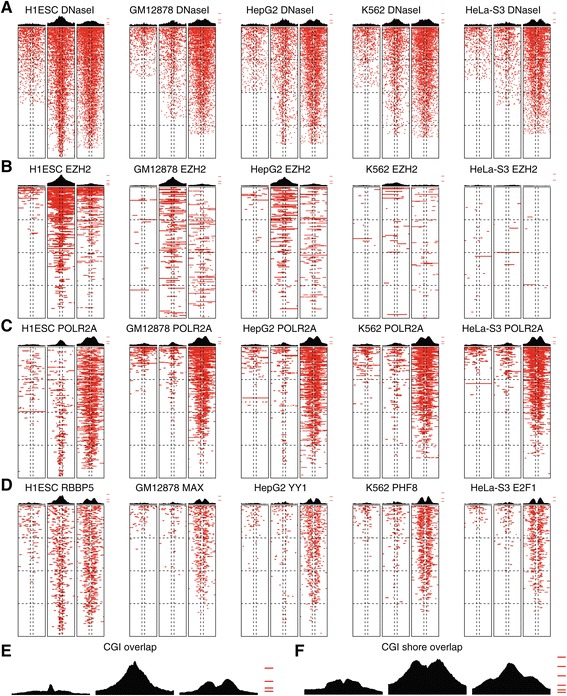
**DHS and RF occupancy at UC DMRs.** Aggregation plots of DHS and RF occupancy are shown for five ENCODE cell types across UC subgroup-specific DMRs stratified into patterns 1 to 3 (left to right for each DHS/RF and cell type combination). Plots are based on 10 kb for each DMR centered on the DMR midpoint. DHS/occupancy across each DMR (rows) is pseudo-colored (red) and DMRs are sorted (vertically, for each DMR pattern separately) according to the number of DHS bases within the 10 kb region. Patterns of DNaseI hypersensitive sites **(A)**, EZH2 **(B)**, POLR2A **(C)**, as well as binding patterns of selected RFs **(D)** pseudo-colored (red) with aggregation plots on top across the different cell lines are shown across the three UC DMR methylation patterns (1 to 3). **(E, F)** Aggregation plots of CGI positions (E) and CGI-shore positions (F) in UC DMRs stratified into patterns 1 to 3. For aggregation plots, tic marks indicate 5%, 10%, 20%, 40%, and 60% basewise occupancy, respectively.

DNaseI hypersensitive sites (DHSs) define regions of open chromatin and are frequently associated with regulatory factor binding. We mapped DHS-peak bases locally in a 10 kb window centered on UC DMRs and explored the spatial patterns of ENCODE ChIP-seq RF binding in relation to DMR positioning. Chromatin accessibility, as measured by DHS, increased towards DMR midpoints and the most frequent UC DMR-DHS overlaps were observed in the H1ESC cell line (Figure 5A). A general trend of decreasing DHS levels across all UC DMR patterns was observed in the more differentiated and cancer-derived-cell lines compared to H1ESC, however this was most accentuated among pattern 2 DMRs (Figure 5A). While pattern 1 DMRs did not exhibit specificity in DHS peak distributions when assessed by aggregation plots (APs), pattern 2 DMRs were centered on DHS sites, and pattern 3 DMRs showed a consistent tendency towards a local depletion of DHSs towards the DMR midpoints. EZH2 binding was strongly associated with DHSs in H1ESC and exhibited a sharp peak centered on pattern 2 DMR midpoints, a feature seen to a lesser extent in GM12878 and HepG2 but lacking entirely in K562 and HeLa-S3 cells (Figure 5B). The observed patterns are consistent with H3K27-trimethylation-mediated repressive/poised state as ‘default’ for pattern 2 DMRs in ESC with a successive transition to stable modes of repression in response to differentiation cues or immortality (Figure 4A). POLR2A binding across pattern 2 DMRs was associated with local DHS density in all cell lines except H1ESC, indicating a decoupling of open chromatin status and gene transcription in ESCs (Figure 5C).

We observed a tendency towards local depletion of POLR2A peak coverage towards pattern 3 DMR midpoints, a feature also observed among a number of RFs across all cell lines and resulting in decidedly bimodal APs (Figure 5C and D). Bimodal AP-plots are commonly the result of non-oriented feature alignments [32]. However, the depletion of RF-binding at pattern 3 DMR midpoints may reflect true features of genome organization as the RF-binding patterns are recapitulated in the patterns of CGI and CGI-shore base overlaps (Figure 5E and F).

### *HOX-*gene silencing in UC exhibits gene expression subtype specificity

**Figure 6:**
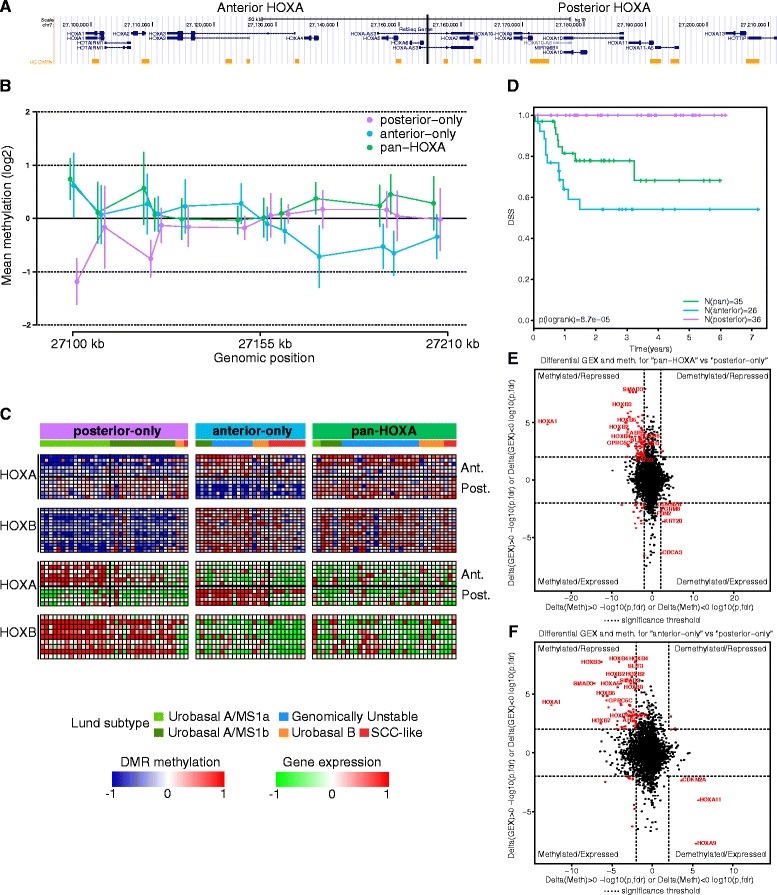
**Methylations patterns in the HOXA locus. (A)** Genomic map of the *HOXA* region with UC DMRs indicated (yellow). **(B)** UC samples were clustered (k-means, k = 3) based on their methylation profiles across the *HOXA*-locus into three groups of samples (posterior-only, anterior-only, and pan-*HOXA*). For each sample group, the average methylation for each DMR is shown across the *HOXA* locus. Vertical bars denote standard deviations. **(C)** DMR methylation and gene expression levels across the *HOXA* and HOXB loci. Within the heatmaps for *HOXA*, the horizontal lines separate the anterior region from the posterior region (between the *HOXA7* and *HOXA9* promoters). The DMRs and genes are in the same order as in panel A and DNA methylation is indicated in blue (low) to red (high) and gene expression in green (low) to red (high). **(D)** Survival analysis of UC samples stratified into the three *HOXA* methylation groups. Disease-specific survival (DSS) was used as endpoint. **(E, F)** Coordinated changes in DMR methylation and gene expression (GEX) were visualized using starburst plots for ‘pan-*HOXA*’ vs. ‘posterior-only’ (E) and ‘anterior-only’ vs. ‘posterior-only’ (F). Dotted lines indicate the significance threshold FDR <0.01 (t-test).

We identified 12 DMRs in the *HOXA*- and 15 DMRs in the *HOXB* locus (Figure 6A), of which a majority exhibited significant negative correlations to mRNA expression. The same effect was observed for a minority of HOXC and HOXD cluster genes. We noticed that the entire *HOXB* locus behaved as one block with respect to DNA methylation and gene expression. Conversely, there was a distinct anti-correlation between the 5’ (posterior) and 3’ (anterior) *HOXA* genes across samples on both the methylation and gene expression levels.

In order to capture the nature of the switch-like appearance and to derive aggregate sample-level *HOXA*-consensus profiles, we performed k-means clustering (k = 3) on all DMRs contained within the *HOXA* locus (Figure 6B). The consensus profiles captured the aggregate structure of the locus and provided readily interpretable methylation patterns. In terms of sample stratification, tumors could be subdivided based on the methylation patterns into those that display ‘posterior-only’, ‘anterior-only’, and ‘pan’ *HOXA* DMR methylation (Figure 6B). A strong link was also observed between anterior *HOXA* (*HOXA1-6*) gene expression and expression patterns across the entire *HOXB*-locus (Figure 6C).

Tumor stratification based on *HOXA*-DMR methylation was also reflected in the global DNA methylation patterns described above. The ‘posterior-only’ group of tumors displayed significantly higher levels of pattern 1 methylation compared to each of the other two *HOX-*methylation-based groups (both *P* <0.001, t-test, FDR corrected). Pattern 2 methylation was significantly higher in both ‘anterior-only’ as well as ‘pan-*HOXA*’ compared to ‘posterior-only’ tumors (both *P* <0.05) but did not differ significantly between the former two, indicating that *HOX-*methylation does not simply recapitulate global methylation patterns. High levels of pattern 3 methylation was characteristic of both ‘anterior-only’ and ‘pan-*HOXA*’ tumors, and differentiated both groups from the tumors displaying ‘posterior-only’ methylation (both *P* <5 × 10^-11^). The absence of a clear difference in pattern 2 methylation suggests that the processes underlying the different epigenetic states within the *HOX-*gene loci are distinct from the ones giving rise to pattern 2 methylation.

With respect to the Lund gene expression subtypes, ‘posterior-only’ tumors corresponded to the Urobasal A gene expression subtype (33/36, *P* <4 × 10^-13^, Fisher’s exact test) and belonged to methylation subgroups 1 and 2 (31/36, *P* <4 × 10^-13^). Within the ‘posterior-only’ tumors, loss of anterior *HOXA* gene expression and increasing posterior *HOXA*-associated DMR methylation was evident and agreed well with the original subdivision of Urobasal A tumors into two subgroups; MS1a (Molecular subtype 1a) and MS1b (Molecular subtype 1b) [4]. Therefore, anterior *HOXA* gene expression is a feature of the MS1a subset of Urobasal A tumors, while the remaining tumors (mainly MS1b) only exhibit sporadic *HOXA* gene expression (Figure 6C). The notion of *HOX-*cluster methylation patterns being related to tumor differentiation states was substantiated by the observation that ‘posterior-only’ tumors were almost exclusively of pathological grade 1 or 2 (34/36). In addition 83% were of pathological stage Ta. ‘Anterior-only’ tumors differed with respect to *HOXA* gene expression patterns. Although near ubiquitous *HOXA9*-expression was the common denominator among these tumors, *HOXA10-13* gene expression was absent in tumors of the Lund SCC-like gene expression subtype of UC (Figure 6C). ‘Anterior-only’ tumors were also enriched for the poor-prognosis SCC-like gene expression subtype of UC (9/13 SCC-like, *P* <0.001) and methylation subgroup 4 tumors (17/27, *P* <0.001). The majority of ‘anterior-only’ tumors (22/27) were of pathological grade 3 while the remaining five were of grade 2, indicating low levels of differentiation. In terms of pathological stage, 21/27 (78%) were invasive (≥T1). ‘Pan-*HOXA*’ tumors were predominantly of the Lund Genomically Unstable subtype (19/35, *P* <0.001) and were weakly enriched for methylation subgroup 3 tumors (14/35, *P* = 0.013). This group of tumors also tended to be invasive (25/35 ≥ T1) and of high grade (23/35 grade 3).

As expected from the Lund subtype as well as clinical associations, the methylation profiles across the *HOXA*-locus stratify the tumors into low- (‘posterior-only’) as well as high-risk (‘pan-*HOXA*’ and ‘anterior-only’) groups in terms of disease-specific survival (Figure 6D, *P* = 8.7 × 10^-5^, logrank test). Thus the coordinated shift in *HOXA/HOXB* loci methylation is strongly associated with a similar shift at the *HOXA/B* expression levels, with genome wide methylation patterns, as well as with previously described molecular (gene expression) subtypes of UC.

### Expression of retinoic acid responsive genes correlates with *HOXA* methylation patterns

The observed pattern of anterior-posterior *HOXA* expression has previously been described in the setting of retinoic acid (RA) induced neuronal differentiation of pluripotent progenitor cells in which undifferentiated cells express the posterior- while repressing the anterior *HOXA*-genes and vice versa [19]. To further investigate the *HOX-*cluster methylation patterns, we performed t-tests for differential methylation and gene expression using the ‘posterior-only’ as a reference group against which ‘anterior-only’ and ‘pan-*HOXA*’ tumors were compared.

The dominant pattern for significant pairs (gene expression and methylation *P* <0.01, FDR corrected) was coordinated increased methylation and reduced gene expression (72/98 in ‘pan-*HOXA’* and 59/76 in the ‘anterior-only’ comparison, Figure 6E and F, Additional file 8: Table S4). In addition to HOX genes *HOXB2-5*, *HOXB8*, and *HOXA1*, additional genes with concomitant gain of methylation and loss of expression in both ‘pan-*HOXA*’ and ‘anterior-only’ tumors included the retinoic acid responsive genes *GPRC5C* [33] and *ITM2B* [34] as well as the transcription factors *SMAD3* and *SLIT3*. A gene significantly methylated and repressed in ‘pan-*HOXA*’ tumors only was *PHF23*, a frequent fusion partner with *NUP98* in acute myeloid leukemia (AML) that has been shown to enforce *HOXA9-10* expression by protecting activating H3K4Me3 marks and blocking EZH2 mediated *HOX-*gene repression [35]. Tumors with ‘pan-HOXA’ methylation patterns also displayed downregulation and methylation of *FABP5*, a key protein in directing the cellular response to RA [36]. Genes exhibiting specific methylation and downregulation in ‘anterior-only’ tumors included additional *HOX-*genes *HOXA5*, *HOXD4*, and *HOXB7* as well as *AHR* which has been shown to modulate retinoic acid receptor/retinoid X receptor (RAR/RXR) mediated cellular responses to RA [37]. Consistent with developmental gene silencing through methylation being the primary factor underlying *HOXA* methylation patterns, few genes exhibited lower methylation levels with concomitant high gene expression levels in either of the two comparisons. Five genes exhibited this pattern for ‘pan-HOXA’ tumors; *CDCA3*, *FBN2*, *GRM8*, *CDKN2A*, and *KRT20*, the latter a marker of terminal urothelial differentiation. For ‘anterior-only’ tumors the same pattern was observed for *HOXA9* and *HOXA11* as well as *CDKN2A*. In addition, we noted that within the ‘posterior-only’ set of tumors, one of the most significantly upregulated genes among tumors expressing the anterior *HOXA* genes (Lund MS1a) versus tumors without anterior *HOXA* expression was *RXRA* (*P* <5 × 10^-7^), providing further evidence in favor of a link between retinoic acid signaling and *HOXA*-gene expression patterns in UC.

### *KDM6A* mutations are depleted in *HOXA9*-expressing tumors

**Figure 7:**
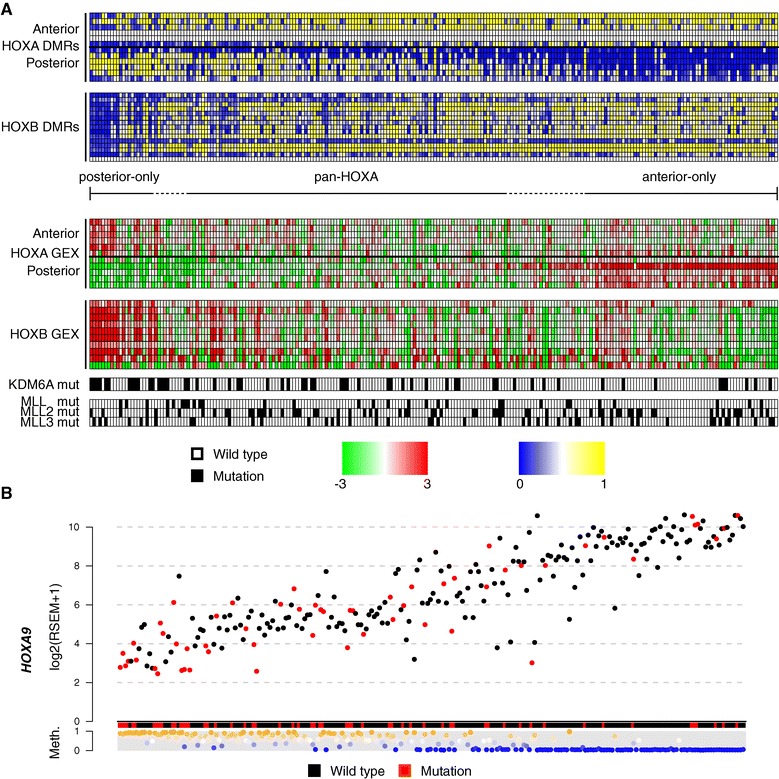
**DNA methylation and gene expression of HOX genes in the TCGA dataset. (A)** Validation of the identified *HOXA* and HOXB DNA methylation and gene expression patterns. DMRs and genes are plotted in genomic 5′ to 3′ order from top to bottom. DNA methylation is indicated in blue (low) to yellow (high) and gene expression in green (low) to red (high). *KDM6A*, *MLL*, *ML2*, and *MLL3* mutation status is indicated below (mutations in black). Within the *HOXA* heatmaps, the horizontal bars separate the anterior region from the posterior region (between the *HOXA7* and *HOXA9* promoters). **(B)** Association between promoter DMR methylation and gene expression for *HOXA9*. Samples are ordered as in (A). *KDM6A* mutations are indicated in red.

We validated our observations on *HOXA/B* cluster methylation and gene expression patterns in external data generated by the TCGA consortium (Methods). Ordering of tumors with respect to the balance of anterior and posterior *HOXA*-DMR methylation validated our observations on the dynamics of *HOX-*gene expression. As the validation dataset only consists of high grade MI tumors, the low-grade associated anterior *HOX* gene expression pattern could only be observed in a small subset of samples, but with retained *HOXB* gene expression as in our own data (Figure 7A).

The trithorax complex and its vertebrate homologs are crucial regulators of homeotic gene expression [38]. The H3K27 demethylase *KDM6A* is among the most frequently mutated genes in UC [14] and its homolog *Utx* has been shown to be a trithorax group regulator in *Drosophila* [39]. In addition, the trithorax complex component *MLL*-genes, encoding H3K4 methyltransferases, are frequently mutated in UC [15]. We therefore investigated the relationship between the *HOXA* methylation subgroups and the trithorax complex linked genes *MLL*, *MLL2*, and *MLL3*, as well as *KDM6A*. While little skewness in MLL-gene mutation rates could be observed, *KDM6A* mutations were depleted in tumors that exhibited unmethylated posterior *HOXA* DMRs (Figure 7A). We also noted that *KDM6A* mutated tumors exhibited significantly lower *HOXA9* gene expression levels (*P* = 0.00035, Wilcoxon rank sum test) and that methylation of the *HOXA9* promoter DMR exhibited a strong negative correlation to gene expression (Figure 7B, Spearmans Rho = -0.78, *P* <2.2 × 10^-16^). In summary, we validate *HOX-*gene methylation- and expression patterns in an independent cohort of MI UC, and highlight a connection between *HOXA9* gene expression patterns and *KDM6A* mutations.

## Discussion

DNA methylation is a multifaceted process with context dependent functions in genome regulation and wide-ranging clinical implications [40,41]. Previous studies of epigenetic alterations in UC have been conducted on low-coverage platforms and have been focused on markers of aggressive disease [10,16,42-44]. Studies that have explored the interrelation of global changes on the epigenetic and gene expression levels have often restricted their analyses to the individual CpG-gene level instead of addressing the associations between global phenotypes [9,11,43,44]. The current study aims at describing the links between gene expression and DNA methylation subtypes of UC as well as investigating the RF binding and chromatin state associations of UC DMRs.

We conducted a comprehensive analysis of differential methylation using MeDIP-chip on 98 UC tumor samples subtyped according to the Lund molecular taxonomy for UC [4]. Bootstrap hierarchical clustering analysis stratified the samples into four subgroups with distinct associations to histopathological groups (stage and grade), mutations (*FGFR3* and *TP53* mutations), as well as Lund gene expression subtypes (Urobasal A, Urobasal B, Genomically Unstable, or SCC-like). The present cluster analysis highlights a clear split between the low grade, non-invasive Urobasal A tumors and the high grade, invasive tumors characterized by genomic instability or a keratinized phenotype (Genomically Unstable and SCC-like tumors, respectively). However, the analysis also revealed that differences in DNA methylation patterns can exist within a group of tumors of the same gene expression phenotype, for example, the presence of Genomically Unstable tumors in methylation subgroups 3 and 4. Importantly we were able to validate our findings in a platform (Nimblegen vs. Illumina) and cohort (Lund vs. TCGA) independent dataset.

Our previous characterization of DNA methylation patterns on low-coverage Illumina 27 K methylation arrays revealed three main methylation subgroups, termed epitypes (A to C) [11]. In the present investigation, subgroup 1 and 2 corresponded to epitype A and exhibited similar histopathological (low pathological stage and grade) and mutational (frequent *FGFR3* and infrequent *TP53* mutations) associations. Subgroup 3 tumors were highly enriched for epitype C tumors, linking this methylation phenotype to the Genomically Unstable gene expression subtype of UC. Finally, subgroup 4 was enriched for epitype B, characterized by extensive demethylation of low CpG density promoter, as well as tumors of the SCC-like gene expression subtype [4,11].

**Figure 8:**
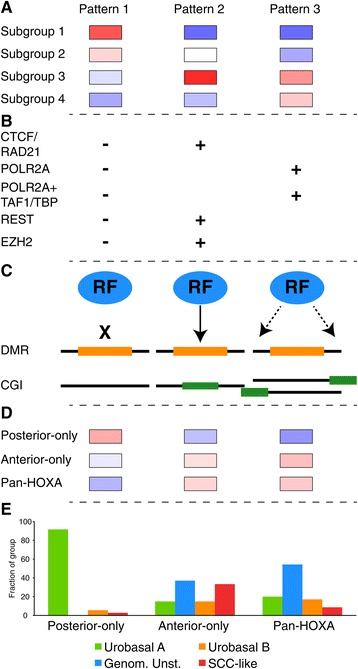
**Schematic summary of genomic associations to UC DMR methylation patterns. (A)** Mean tumor subgroup methylation levels with respect to DMR methylation patterns. Methylation levels are indicated in blue (low) to red (high). **(B)** Pattern 1 to 3 DMRs display differential binding of regulatory factors in ENCODE cell lines. **(C)** A model of modes of RF binding at pattern 2 and pattern 3 DMRs, indicating differential regulatory function or modular organization underlying differential methylation. **(D)** Mean pattern 1 to 3 methylation levels in tumors belonging to the different *HOXA* methylation groups. **(E)** Lund gene expression subtype distribution across the *HOXA* methylation groups.

Previous studies into epigenetic changes in UC have mainly been focused on characterizing differential methylation [11,16,42-44]. However, the functional genomic context of differential methylation remains less well studied. We used multi-level genomic data generated through the ENCODE consortium to characterize the regulatory potential of UC DMRs and show that the identified regions exhibit biologically coherent chromatin state and RF-binding preferences in ENCODE cell-lines. We found that subgroup-defining DMRs exhibit three distinct patterns of methylation across tumors (summarized in Figure 8A). Pattern 1 DMRs are located in low CpG-density, repeat-rich, subtelomeric regions of the genome and are depleted of functional chromatin states and RF-binding across ENCODE cell lines. Methylation of pattern 1 DMRs is inversely correlated with pathological grade and may represent stochastic demethylation of heterochromatic DNA through a loss of a maintenance-like process, or may be a product of the formation of partially methylated domains (PMDs) [45] in a subset of tumors. The implications of subtelomeric and repetitive sequence demethylation for genome stability are not well understood but may contribute to UC pathogenesis and disease progression.

*De novo* methylation of high CpG-density positions is a common feature of an aggressive subset of UC tumors [11,16]. Pattern 2 DMRs are enriched for conserved, high CpG-density (CGI), repeat-depleted, regions marked by bivalent domains in embryonic stem cells. This pattern of DMR methylation does not correlate with gene expression, is present in a subset of high grade tumors and is tightly linked to the Genomically Unstable gene expression subtype of UC. We identified pattern 2 DMRs as sites of EZH2 and REST binding, as well as CTCF/RAD21 binding, in H1ESC (summarized in Figure 8B). EZH2 is a core component of PRC2 that mediates polycomb silencing of developmental genes [23,24,27,46]. REST is involved in repression of differentiation associated genes in the neural lineage, is essential for embryonic development [47] and has been implicated in the process of carcinogenesis [48,49]. Evidence for a direct role for DNA methylation in NRSF/REST mediated gene suppression has also been reported [50] and a connection between REST binding and polycomb mediated gene repression has been established [51]. The CTCF/RAD21 binding patterns may implicate disruption of cohesin function as either the cause or consequence of cluster 2 methylation at a subset of UC DMRs. Whereas a large proportion of CTCF/RAD21 marked sites were devoid of additional RF binding, a subset displayed near ubiquitous POLR2A and RF binding with accompanying active marks in the four cell lines for which chromatin tracks were available. The observed patterns may reflect different modes of cohesin involvement in gene regulation [52]. In support of a functional role for differential methylation at sites of CTCF and cohesin co-localization, Parelho *et al.* have shown that differential DNA methylation of CTCF motifs at cell type specific cohesin sites can abrogate CTCF mediated cohesin binding [31]. As we found specific enrichment of CTCF/RAD21 colocalization across pattern 2 DMRs, this methylation pattern may identify a subset of tumors with actionable defects in cohesin function [53]. Our findings link pattern 2 methylation to developmental gene silencing as well as disruption of factors mediating higher-order chromatin structure.

Pattern 3 DMRs were enriched for CGI shore overlaps and captured the dynamic regulatory nature of DNA methylation in terms of identifying sequences associated with active and gene-regulatory chromatin states as well as transcription and enhancer function related RF-binding. These findings are in line with observations on tissue-specific CpG island shore methylation in colorectal cancer [54]. With respect to H1ESC polycomb marks, pattern 3 DMRs displayed a wider range of active chromatin marks across eight cell lines with a more differentiated phenotype than did pattern 2 DMRs. Methylation at pattern 3 DMRs was also frequently correlated with gene expression. These findings are consistent with the notion of pattern 3 methylation being involved in active gene regulation. Pattern 2 and pattern 3 DMRs differed with respect to the spatial binding of regulatory factors in five ENCODE cell lines. Whereas ChIP-seq binding peaks were centered on pattern 2 DMRs, pattern 3 DMRs exhibited a marked depletion of RF-binding. This points towards differential regulatory function or modular organization at sites of pattern 2 and 3 DMRs (Figure 8C). An interpretation of RF binding at pattern 3 DMRs with respect to CpG-density is that RF binding occurs in regions of elevated CpG-density, with coordinated methylation changes in adjacent lower CpG-density regions. Consistent with this notion, tissue-specific DMRs associated with developmental processes tend to overlap CGI shores [55]. Pattern 2 DMRs exhibit high CpG densities and display lower regional DNaseI hypersensitivity and RF occupancy in non-ES cell lines. Whereas methylation at pattern 2 DMRs may represent a more permanent inactivation with subsequent heterochromatinization of developmental gene loci, pattern 3 DMR methylation may serve as a dynamic readout of local transcriptional activity.

We characterize a switch-like pattern, previously unreported in the context of UC, involving the anterior and posterior *HOXA* as well as the entire *HOXB* locus. The switch-like pattern is likely a consequence of differential activation of conserved topologically associating domains (TADs) that divide the *HOXA*-locus into separate regulatory units [56]. TAD boundaries are frequently marked by CTCF binding, and the *HOXA5* promoter DMR that demarcates the switching-point overlaps ENCODE CTCF ChIP-seq binding peaks. The epigenetic states of *HOX-*gene promoter DMRs are reflected in the mRNA expression patterns and are associated with tumor grade. We therefore hypothesize that *HOX-*gene expression patterns in UC may reflect the differentiation competency or state of the tumor cells. In support of this conclusion, a similar epigenetic switch has been described in the context of RA-induced differentiation in the non-malignant setting [19]. Multiple RA-responsive genes exhibited coordinated changes in promoter methylation and mRNA expression with respect to the different patterns of *HOX*-gene expression, consistent with RA being a crucial mediator of urothelial differentiation [57,58]. Anterior *HOXA*-gene silencing has previously been observed in the context of multiple regional epigenetic silencing and was shown to identify a poor-prognosis subgroup of UC [10]. The study did not however report on the switch-like behavior of anterior and posterior *HOXA*-genes present in a subset of high grade tumors allowing further stratification of the poor-prognosis group. The mechanisms of differential activation of the posterior and anterior *HOXA* TADs in UC have not been explored, but may provide insight into the processes underlying tumor differentiation states. In this context, a differential response to all-trans retinoic acid (ATRA) or demethylating agents in tumors with ‘anterior-only’ or ‘pan-*HOXA*’ methylation patterns could be clinically significant.

With respect to tumor stratification the two schemes based on *HOX-*gene and global DMR methylation respectively exhibited broad commonalities. While the low stage and grade subgroup 1 and 2 tumors corresponded to the ‘posterior-only’ group, subgroup 3 tumors only exhibited a slight bias towards the ‘pan-*HOXA’* group. Subgroup 4 included the majority of SCC-like tumors as did the ‘anterior-only’ group. Global DNA methylation patterns were however only moderately captured by the *HOX-*based stratification arguing in favor of separate mechanisms underlying global- and HOX-locus methylation patterns (Figure 8D). The finding that tumors of a given gene expression subtype can express different sets of *HOX-*genes was particularly evident for the Genomically Unstable subtype (Figure 8E) and could indicate that the same aggregate gene-expression phenotype can be reached through different paths. Alternatively, expression of different sets of *HOX-*genes may reflect a positional identity as differential *HOX-*gene expression within the genitourinary system has been described previously [59,60], or be a readout of the local balance of developmental morphogen signaling.

We were also able to provide independent validation of *HOX-*gene silencing patterns in TCGA data and highlight a potential connection between the absence of *KDM6A* mutations and posterior *HOXA* methylation patterns. The most prominent driver of the three cluster split in the validation set was the *HOXA9*-promoter-associated DMR. Methylation of the *HOXA*9 DMR was incompatible with *HOXA9* expression, although an unmethylated state did not strictly translate into expression. KDM6A modulates *HOX-*gene expression through removal of H3K27Me3-marks [61,62] and exhibits differential *HOX-*gene occupancy patterns with respect to cellular origin as well as differentiation states [62]. Although *KDM6A* mutations were not mutually exclusive with any of the posterior *HOXA* expression patterns, further investigations into differential *HOX-*gene expression patterns are warranted in light of confounding factors such as tumor heterogeneity and gene functional redundancy. Inactivation of polycomb-related epigenetic modifiers through gene mutations are likely early events in UC formation [13,14]. The selection pressures and processes leading to differential mutation and epigenetic landscapes across tumor subgroups are however unknown. Inquiries connecting the developmental biology of the bladder with the tumor biology of UC are beginning to provide insight into these basic questions [57,63-65] and future investigations should be directed at understanding epigenetic changes in the context of molecular subgroups and underlying biological processes.

## Conclusions

In summary, we leverage multi-level genomic data to characterize regions of the genome associated with differential methylation in UC. We provide insight into the functional genomic context underlying differential methylation, validate our findings with independent data, and describe novel connections between the epigenetic, genetic, and phenotypic levels in UC. Our current work integrates ENCODE data and connects distinct features of the genome to three broad methylation patterns with strong phenotypic associations. Finally, we characterize a putative actionable epigenetic switch involving *HOX-*genes with strong correlations to tumor differentiation states and propose that a link exits between *KDM6A* mutations and *HOXA9* gene expression patterns.

## Additional files

Additional file 1: Methods. This file contains an extended methods description. Additional file 2: Table S1. List of TCGA-data files used for the validation analyses. Additional file 3: Table S2. Genomic coordinates (hg18) of all 5,453 UC DMRs identified in the present study with genomic feature and gene expression correlation annotations. Additional file 4: Figure S1.
**(A)** Differences in CpG/bp between DMRs with and without subtype-specific methylation patterns. **(B)** Differences in CpG/bp between hyper- and hypomethylated (absolute M >0.25) subtype-specific DMRs. Additional file 5: Figure S2. Mean methylation levels across methylation subgroups for pattern 1 **(A)**, pattern 2 **(B)**, and pattern 3 **(C)** DMRs. Additional file 6: Figure S3.
**(A)** Number of DMRs with local LINE1 or LTR repetitive elements. DMR localization in subtelomeric regions. Visualization in boxplot **(B)** and 1 Mb bin format **(C)**, respectively. Additional file 7: Table S3. Top results of MSigDBv3.1-signature analysis with respect to DMR methylation patterns. Additional file 8: Table S4. Significantly correlated DMR-gene expression pairs for the ‘posterior-only’ vs. ‘anterior-only’ and ‘posterior-only’ vs. ‘pan-HOXA’ analyses.

## Abbreviations

Bp: Basepair
CGI: CpG island
ChIP: Chromatin immunoprecipitation
DHS: DNaseI hypersensitive site
DMR: Differentially methylated region
kb: Kilo basepair
MeDIP: Methylated DNA immunoprecipitation
MI: Muscle invasive
MS: Molecular subtype
NCEC: Non-coding evolutionarily conserved
NMI: Non muscle-invasive
PRC2: Polycomb repressive complex 2
RA: Retinoic Acid
RF: Regulatory factor
SCC-like: Squamous cell carcinoma-like
TCGA: The cancer genome atlas project
UC: Urothelial carcinoma
