# Supplementary material for: Integrative epigenomic analysis of differential DNA methylation in urothelial carcinoma
Source: Genome Med. 2015 Mar 10;7(1):23. doi: 10.1186/s13073-015-0144-4 (PMC4373102; doi:10.1186/s13073-015-0144-4)
Supplement: Additional file 1: Methods. — This file contains an extended methods description. [file 13073_2015_144_MOESM1_ESM.pdf]

Supplemental Appendix

Supplemental Methods

Tumor samples

Cold cup biopsy samples from patients undergoing transurethral resection within the South Sweden Health Care Region were obtained as part of a routine sample collection effort in collaboration with the urological department at Skåne University Hospital. All tumor samples were re-evaluated for stage and grade by an experienced pathologist and clinical follow-up data was obtained in collaboration with the department of urology at Lund University. Four normal urothelium samples included in the present study were obtained by macrodissection from patients undergoing surgery for localized prostate cancer. Nucleic acid extraction (DNA and RNA) had previously been performed as described in [1]. The starting point for the sample selection process was a set of 308 UC tumors for which gene expression data on the Illumina HT-12 platform (Illumina, San Diego, USA) was available (GSE32894) [1]. A random subset of the 308 tumors (N=149) had previously been run on low-coverage Illumina Methylation 27K arrays and shown to exhibit high tumor cell contents [2]. We used information on both the gene expression- and when available DNA methylation level to exclude samples showing evidence of extensive immune cell infiltration or low tumor cell content i.e. Lund “Infiltrated” and Lauss et al. 2012 “epitype D” tumors [1, 2]. Tumors belonging to the SCC-Like subgroup of UC differ distinctly from “Infiltrated“ tumors on the gene expression level, but cluster with epitype D tumors on the methylation level. Seven epitype D/SCC-Like tumors were included in the present study. Ultimately, 98 tumor samples representative of all major gene expression and DNA-methylation subtypes previously defined by us were selected for MeDIP-based methylation analysis. Data on *TP53* and *FGFR3* mutation status was available for all tumors included in the study [3].

Methylated DNA immunoprecipitation and array hybridization

For each sample a total of 1500ng of genomic DNA in a volume of 60ul TE-buffer (Promega, Fitchburg, USA) was subjected to sonication using the Bioruptor UCD-200 system (Diagenode, Liege, Belgium) in batches of six samples, yielding a fragment range (250-500bp) suitable for MeDIP [4] and array hybridization. The fragment distribution was assessed by agarose gel electrophoresis (Invitrogen, Carlsbad, USA) of 250ng of sonicated DNA and quantitation using ImageJ software [5]. Methylated DNA immunoprecipitation was performed in batches of eight samples using the MagMeDIP-kit (Diagenode, Liege, Belgium) and purified using the iPure-kit (Diagenode, Liege, Belgium) according to manufacturers instructions. Each MeDIP-reaction included an in vitro methylated and demethylated arabidopsis DNA spike-in control (Diagenode, Liege, Belgium). Specific enrichment of methylated DNA in the antibody versus input control fraction was verified using qPCR for the two spike-in controls as well as the endogenous genes TSH2B (positive) and GAPDH (negative).

Following MeDIP and purification, the enriched as well as input fractions of each sample were amplified using the GenomePlex Complete Whole Genome Amplification Kit (Sigma-Aldrich, St. Louis, USA) and purified using the QIAquick PCR Purification Kit (QIAGEN, Venlo, Netherlads). All primary amplification products (input and enriched fractions) were run on agarose gels to verify a suitable fragment range and then re-amplified using the GenomePlex WGA Reamplification Kit (Sigma-Aldrich, St. Louis, USA), yielding sufficient amounts of high quality DNA for array hybridization. The purified amplification products were processed, labeled, and hybridized to NimbleGen Human DNA Methylation 3x720K CpG Island Plus RefSeq Promoter Arrays (Roche Nimblegen, Madison, USA) by the NimbleGen genomics facility on Iceland (www.nimblegen.com) and the resulting raw files containing probewise intensity data for the enriched (Cy5) and input (Cy3) channels were obtained from the company. In order to minimize the downstream influence of batch effects, the samples were randomized to sonication batches initially and re-randomization was performed between each major subsequent step, i.e. MeDIP,

re-amplification and hybridization in order to allow for identification and removal of variation attributable to technical artefacts.

Data filtering, normalization, and variance-based detection of DMRs

The data was normalized using a five-step normalization scheme which consisted of 1) lowess normalization of the log2-transformed individual dye channels with respect to probe GC-content, 2) forming of ratios by subtracting the input channel intensity from the enriched channel intensity for each sample, 3) quantile normalization of data across all samples, 4) median merging of duplicate probes, 5) application of a principal component analysis based method for identification and removal of variation related to technical factors such as sonication- and MeDIP batches [6]. The normalized and merged data set consisted of 676763 probes for each of the 102 samples included in the downstream analyses.

Between-tumor DMRs were defined using custom R-scripts. Observed variances were calculated for all array probes by calculating the pooled variance of probes within a 500 bp window. In order to evaluate the significance of the observed variances, a null distribution was formed for each window by randomly sampling from the distribution of variance meassurements and recalculating the pooled variance 25000 times. To account for effects on variance due to local CpG density, the resampled values were drawn from CpG density matched bins. The probe-CpG coupling factor [7], rounded to the nearest whole integer, was used as a meassure of local CpG density. For each 500 bp window, the 99th percentile of the generated null distribution was chosen as the significance threshold for calling DMRs. In cases where two or more significant windows had any degree of sequence overlap, the DMR borders were extended outward until the percentile scores of the outermost probes dropped below the chosen significance threshold.

Clustering of tumor samples and DMRs

Hierarchical clustering (Pearson distance, Ward's algorithm) of a bootstrapped data set with respect to tumors was performed 10000 times. For each iteration, the hierarchical tree was cut in a fashion yielding k clusters and the cluster assignment of each tumor was recorded for each iteration. The resulting coclustering matrix was in turn clustered and cut at the level yielding k clusters, and providing subgroup assignents for each tumor. A k=4 split using Pearson distance showed optimal sample stratification and was therefore chosen. In order to define DMRs with subgroup-specific methylation patterns, we applied ANOVA to all 5453 UC DMRs. The raw ANOVA p-values were corrected for multiple testing using the fdr method [8] and DMRs with an adjusted p-value below 0.05 (N=2697, 49.5%) were considered as having subgroup specific methylation patterns. Hierarchical clustering of subgroupe-specific DMRs was performed using Pearson distance and Wards algorithm. The resulting dendrogram was cut at the three-branch level, yielding the DMR methylation patterns 1-3. Sample clustering based on *HOX*-gene methylation patterns was carried out by k-means clustering of the DMRs located within the *HOXA*-locus (N=12). The k-means algorithm was initialized 1000 times and the clustering output of the iteration with the greatest percent variance explained metric was chosen. The most stable iteration classified 36 samples as "posterior-only", 27 as "anterior-only" and 35 as "pan-*HOXA*".

Annotation of genomic features to DMRs

For the RefSeq gene track, the exon structures of all transcripts having the same gene symbol, orientation and transcription start site were merged. Promoter regions were defined as bases -2000 to +500 relative the transcription start site of the merged RefSeq gene models. The gene models were annotated to all DMRs that had any degree of promoter or gene body overlap, i.e. DMRs could be associated to multiple gene symbols. Data on CpG island localization was obtained from the UCSC genome browser (genome.ucsc.edu) and CGI shores were defined as 2000 basepairs up- and downstream of islands [9]. Basewise CGI and shore overlaps with UC

DMRs were calculated. Above 50% DMR sequence overlap with the CGI bases was used as a cutoff for calling overlaps. Repetitive elements belonging to the L1 family of LINEs as well as LTR elements were lifted from the RepeatMasker track and basewise overlaps with UC DMRs were calculated for each element type and DMR separatelly.

Evolutionarily constrained element overlaps were determined by parsing the list of GERP elements so that all elements having any degree of overlap with exonic RefSeq bases were filtered out. Overlaps between the remaining elements and UC DMRs were quantified and in cases were multiple constrained elements were within a DMR, the total number of constrained bases was recorded. A cutoff of 10 percent sequence overlap, with conserved elements was chosen and each DMR was classified as above 10% conserved or not. The conclusions in terms of effect sizes and significances would however have remained the same if a cutoff level of e.g. 25% or 200bp would have been chosen.

The PRC2 target signature was obtained from Lee et al. [10] and gene symbols of PRC2 marked genes were matched to the RefSeq gene model annotations of the UC DMRs. Chromatin tracks for nine ENCODE cell lines [11] were obtained using the UCSC genome browser and basewise overlaps between UC DMRs and the 15 different states were quantified within each cell line. For each cell line, a consensus state was assigned by majority vote to all DMRs. In cases of ties, an arbitrary tiebreak was applied. To quantify the local distribution of chromatin states at RefSeq transcription start sites and UC DMRs, basewise sequence overlaps between chromatin states were quantified in a 4kb window.

For assignment of biological processes to UC DMRs the MSigDB v3.1 database (broadinstitute.org/gsea) was downloaded and the gene signatures were extracted. The full set of RefSeq gene symbols used to annotate the UC DMRs was used as the background and

signature enrichments for the DNA methylation clusters were calculated using Fishers exact test with fdr correction for multiple testing [8].

Gene expression data processing and analyses

Following normalization of the raw expression data (see [1]), a prefiltering step was carried out to eliminate all probes without an annotated RefSeq transcript model. For the remaining probes a 25% intensity filter followed by median merging on gene symbol and mean centering of rows, yielded a matrix with expression data for 19843 unique genes. The gene expression data was matched to DMR RefSeq annotations and the association between DNA methylation and gene expression determined using Pearson correlation. To derive empirical cutoffs for calling significant correlations we permuted the rows of the DMR-matched gene expression data set 1000 times and recalculated the correlations to the methylation data. The 2.5 and 97.5 percentiles of the resulting distribution were used as cutoffs for calling significant correlations (cutoffs $r>0.2467$ or $r<-0.2486$), each DMR was assigned the best correlation coefficient in cases where multiple genes with available expression data overlapped it.

TCGA data validation

Stable methylation subgoups of UC were derived by applying consensus clustering [12] to the 2000 most varying probes across the full Illumina methylation 450K data as well as the top 25 percent most varying UC DMR overlapping probes (N=841) using the "ConsensusClusterPlus" package in R [13]. Briefly, kmeans clustering was performed 2000 times with parameters clusterAlg="km", maxK=6, pItem=.8 and pFeature=1. The resulting coclustering frequency matrices were converted to dissimilarity metrics and clustered hierarchically using the average linkage method. The resluting hierarchical trees were cut at the level yielding k subgroups and providing each sample with a consensus cluster designation for all k (k=1,2,..,6). Results were evaluated for all k using plots generated by the clustering function as well as by visual inspection

of subgroup heatmap profiles. Clustering of heatmap rows was performed using Pearson distance and Ward's linkage algorithm. For validation of HOX-gene methylation patterns, the *HOXA*-DMR overlapping probes were extracted from the full data matrix and the most varying probe was retained for each unique *HOXA*-DMR (data available for 10/12 DMRs). For visualization purposes (Figure 7), the samples were rank-ordered based on the balance of anterior and posterior *HOXA* gene expression using the formula: mean(*HOXA1-6*)+mean(*HOXB*)-*HOXA9*-mean(*HOXA10-13*).

Processing and analysis of ENCODE regulatory factor binding data

The file "wgEncodeRegTfbsClusteredWithCellsV3.bed", containing hg19 mapped RF binding peak calls derived from 690 ChIP-seq data sets representing 91 cell lines and 161 unique factors generated by ENCODE [14], was processed to separate all factor-cell line combinations. The five cell lines for which the greatest number of RF's had been run (K562, GM12878, HepG2, HeLa S3 and H1ESC. All N≥50) were selected and basewise overlaps with UC DMRs in a 10001 bp window were obtained using the "GenomicRanges" package in R. A binary matrix of strict DMR-RF overlaps was constructed by identifying all instances in which a UC DMR had any degree of sequence overlap with a RF binding peak. For clustering of UC DMRs based on RF-binding, the overlap of factors run in all cell lines (N=18 unique factors per cell line) was extracted and all DMRs with a minimum of one bindinig instance (N=3014) were retained. The rows and columns of the binary overlaps matrix were hierarchically clustered using Ward's algorithm and Jaccard distance as the dissimilarity metric and visualized in annotated heatmap format using R. Specific enrichment of RF overlaps with the four previously defined DMR clusters was calculated in the following way; for single factors (e.g. EZH2) any binding peak overlap in a cell line was recorded, for combinations of factors (e.g. CTCF+RAD21), a DMR was assigned an overlap only if all factors had overlapping peaks within the same cell line. From the UCSC genome browser, we obtained peak calls for DNaseI hypersensitive sites across the five

ENCODE cell lines (uniform peaks pipeline) as well as RefSeq CGI calls. We quantified basewise overlaps in a 10 kb window centered on UC DMRs. For the three patterns of DMRs, we plotted the profiles of DHS's and RF's across the five cell lines in relation to UC DMR midpoints in the 10 kb window for which overlaps had been extracted. For all instances in which multiple 10kb windows overlapped, one DMR was selected at random and the final plots included data for 4496 DMRs. For visualization and comparability purposes, the plotting window for each DMR pattern was scaled to unit length. The mean basewise binding profile was caluculated for each of the 3 DMR patterns and the range of the profile plots was set to the the percentage of the highest coverage base plus a 10% pad. Within each DMR pattern, the rows were ordered based on the total number of DNaseI HS bases within the window. Enrichment and depletion statistics reklated to RF binding were derived using Fisher's exact test and fdr correction for multiple testing applied when the number of assayed RF-cell line combinations exceeded 5.

Supplemental Figure Legends

Supplemental Figure 1.

A) Differences in CpG/bp between DMRs with- and without subtype-specific methylation patterns. **B)** Differences in CpG/bp between hyper- and hypomethylated (absolute M>0.25) subtype-specific DMRs.

Supplemental Figure 2. Mean methylation patterns across methylation subgroups for pattern 1 **(A)**, pattern 2 **(B)** and pattern 3 **(C)** DMRs.

Supplemental Figure 3. A) Number of DMRs with local LINE1 or LTR repetitive elements. DMR localization in subtelomeric regions. Visualization in boxplot- **(B)** and 1Mb bin format **(C)** respectively.

Supplemental Tables

Supplemental table 1

List of TCGA-data files used for the validation analyses.

Supplemental table 2

Genomic coordinates (hg18) of all 5453 UC DMRs identified in the present study with genomic feature and gene expression correlation annotations.

Supplemental table 3

Top results of MSigDBv3.1-signature analysis with respect to DMR methylation patterns.

Supplemental table 4

Significantly correlated DMR-gene expression pairs for the ”posterior-only” vs ”anterior-only”and ”posterior-only” vs ”pan-HOXA” analyses.

Supplemental References

1. Sjodahl G, Lauss M, Lovgren K, Chebil G, Gudjonsson S, Veerla S, Patschan O, Aine M, Ferno M, Ringner M, et al: **A molecular taxonomy for urothelial carcinoma.** *Clinical cancer research : an official journal of the American Association for Cancer Research* 2012, **18:**3377-3386.
2. Lauss M, Aine M, Sjodahl G, Veerla S, Patschan O, Gudjonsson S, Chebil G, Lovgren K, Ferno M, Mansson W, et al: **DNA methylation analyses of urothelial carcinoma reveal distinct epigenetic subtypes and an association between gene copy number and methylation status.** *Epigenetics : official journal of the DNA Methylation Society* 2012, **7:**858-867.
3. Sjodahl G, Lauss M, Gudjonsson S, Liedberg F, Hallden C, Chebil G, Mansson W, Hoglund M, Lindgren D: **A systematic study of gene mutations in urothelial carcinoma; inactivating mutations in TSC2 and PIK3R1.** *PloS one* 2011, **6:**e18583.
4. Weber M, Davies JJ, Wittig D, Oakeley EJ, Haase M, Lam WL, Schubeler D: **Chromosome-wide and promoter-specific analyses identify sites of differential DNA methylation in normal and transformed human cells.** *Nature genetics* 2005, **37:**853-862.
5. Schneider CA, Rasband WS, Eliceiri KW: **NIH Image to ImageJ: 25 years of image analysis.** *Nature methods* 2012, **9:**671-675.
6. Lauss M, Visne I, Kriegner A, Ringner M, Jonsson G, Hoglund M: **Monitoring of technical variation in quantitative high-throughput datasets.** *Cancer informatics* 2013, **12:**193-201.

7. Down TA, Rakyan VK, Turner DJ, Flicek P, Li H, Kulesha E, Graf S, Johnson N, Herrero J, Tomazou EM, et al: **A Bayesian deconvolution strategy for immunoprecipitation-based DNA methylome analysis.** *Nature biotechnology* 2008, **26:**779-785.
8. Benjamini Y, Hochberg Y: **Controlling the False Discovery Rate - a Practical and Powerful Approach to Multiple Testing.** *J Roy Stat Soc B Met* 1995, **57:**289-300.
9. Irizarry RA, Ladd-Acosta C, Wen B, Wu Z, Montano C, Onyango P, Cui H, Gabo K, Rongione M, Webster M, et al: **The human colon cancer methylome shows similar hypo- and hypermethylation at conserved tissue-specific CpG island shores.** *Nature genetics* 2009, **41:**178-186.
10. Lee TI, Jenner RG, Boyer LA, Guenther MG, Levine SS, Kumar RM, Chevalier B, Johnstone SE, Cole MF, Isono K, et al: **Control of developmental regulators by Polycomb in human embryonic stem cells.** *Cell* 2006, **125:**301-313.
11. Ernst J, Kheradpour P, Mikkelsen TS, Shoresh N, Ward LD, Epstein CB, Zhang X, Wang L, Issner R, Coyne M, et al: **Mapping and analysis of chromatin state dynamics in nine human cell types.** *Nature* 2011, **473:**43-49.
12. Monti S, Tamayo P, Mesirov J, Golub T: **Consensus clustering: A resampling-based method for class discovery and visualization of gene expression microarray data.** *Mach Learn* 2003, **52:**91-118.
13. Wilkerson MD, Hayes DN: **ConsensusClusterPlus: a class discovery tool with confidence assessments and item tracking.** *Bioinformatics* 2010, **26:**1572-1573.
14. The ENCODE Project Consortium: **An integrated encyclopedia of DNA elements in the human genome.** *Nature* 2012, **489:**57-74.
