# Supplementary figures and images for: Integrative epigenomic analysis of differential DNA methylation in urothelial carcinoma
Source: Genome Med. 2015 Mar 10;7(1):23. doi: 10.1186/s13073-015-0144-4 (PMC4373102; doi:10.1186/s13073-015-0144-4)

**A**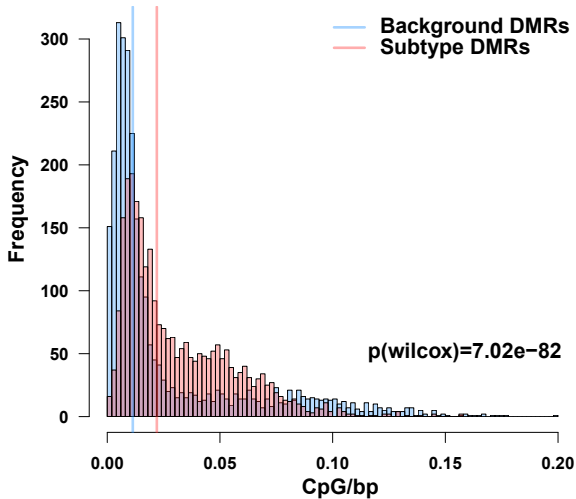**B**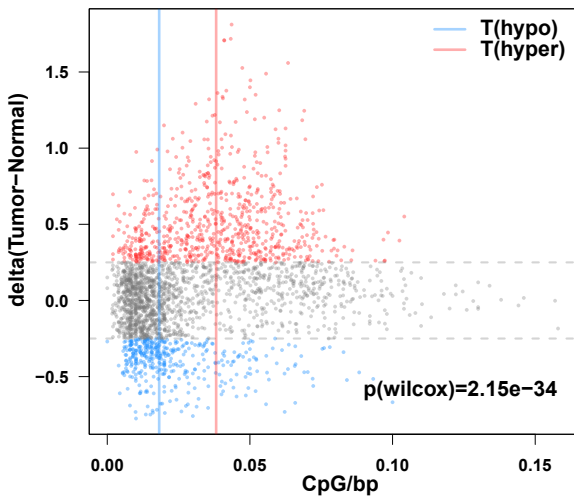

Supplement: Additional file 4: Figure S1. — (A) Differences in CpG/bp between DMRs with and without subtype-specific methylation patterns. (B) Differences in CpG/bp between hyper- and hypomethylated (absolute M >0.25) subtype-specific DMRs. [file 13073_2015_144_MOESM4_ESM.pdf]

**A**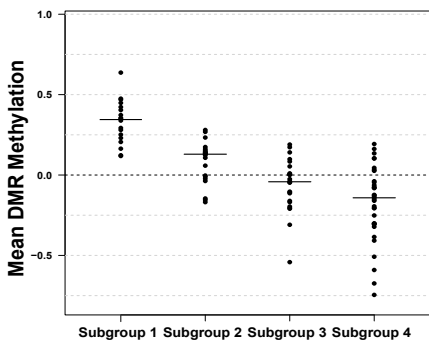**B**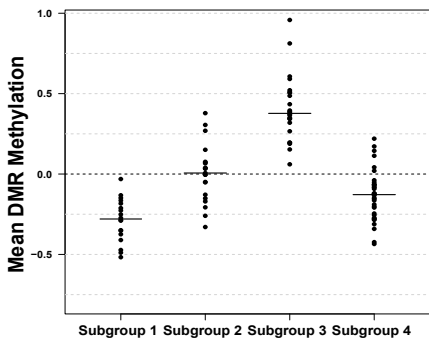**C**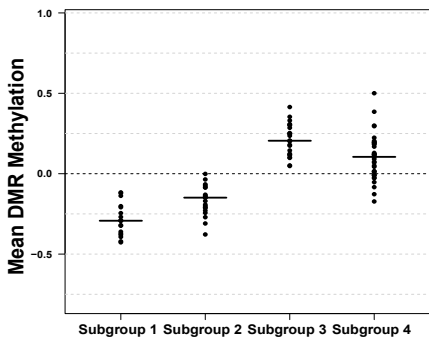

Supplement: Additional file 5: Figure S2. — Mean methylation levels across methylation subgroups for pattern 1 (A), pattern 2 (B), and pattern 3 (C) DMRs. [file 13073_2015_144_MOESM5_ESM.pdf]

**A**

|            | LINE1/LTR overlap |            |
|------------|-------------------|------------|
|            | False (%)         | True (%)   |
| Background | 1825 (66.2)       | 931 (33.8) |
| Pattern 1  | 461 (68.6)        | 211 (31.4) |
| Pattern 2  | 583 (89.7)        | 67 (10.3)  |
| Pattern 3  | 1062 (77.2)       | 313 (22.8) |

**B**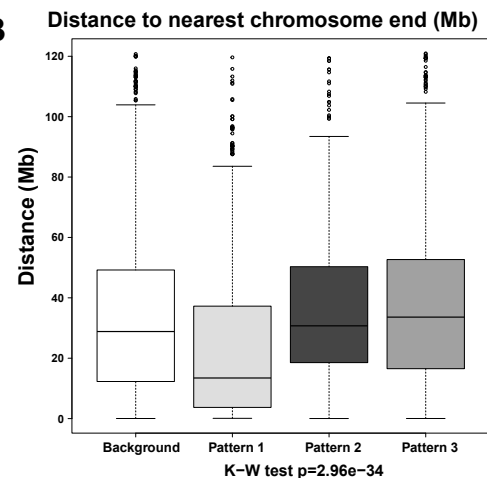**C**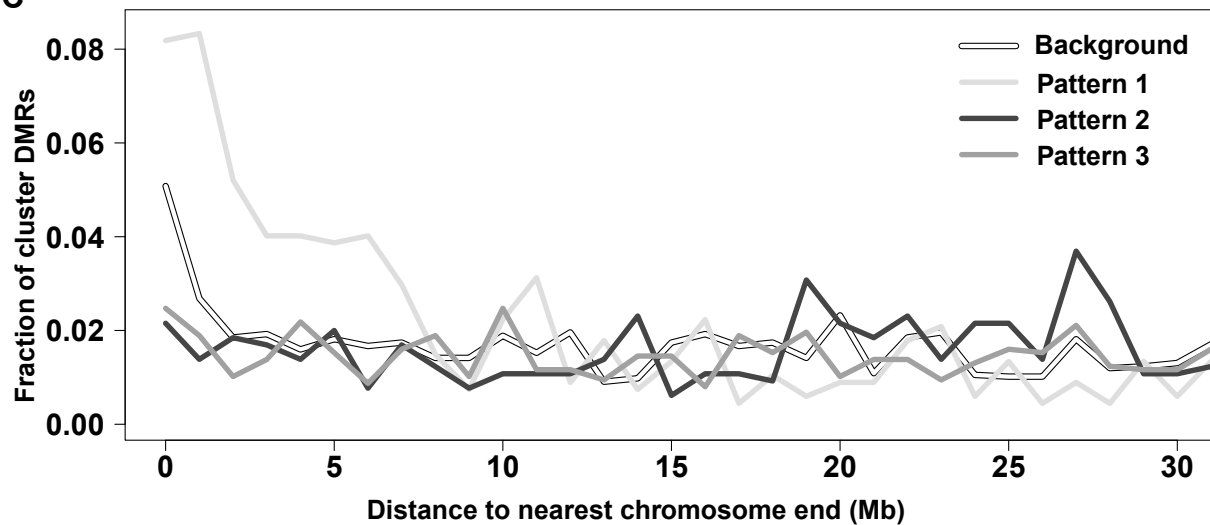

Supplement: Additional file 6: Figure S3. — (A) Number of DMRs with local LINE1 or LTR repetitive elements. DMR localization in subtelomeric regions. Visualization in boxplot (B) and 1 Mb bin format (C), respectively. [file 13073_2015_144_MOESM6_ESM.pdf]
